# Eddy covariance measurement-based differences in annual evapotranspiration between forests and grasslands in China

**DOI:** 10.3389/fpls.2022.1030929

**Published:** 2022-11-25

**Authors:** Renxue Fan, Mingyu Sun, Xianjin Zhu, Qiufeng Wang

**Affiliations:** ^1^ College of Agronomy, Shenyang Agricultural University, Shenyang, China; ^2^ School of Earth System Science, Tianjin University, Tianjin, China; ^3^ Synthesis Research Center of Chinese Ecosystem Research Network, Key Laboratory of Ecosystem Network Observation and Modeling, Institute of Geographic Sciences and Natural Resources Research, Chinese Academy of Sciences, Beijing, China; ^4^ College of Resources and Environment, University of Chinese Academy of Sciences, Beijing, China

**Keywords:** evapotranspiration, eddy covariance, spatial variability, climate change, vegetation area

## Abstract

Annual evapotranspiration (AET), the total water vapor loss to the atmosphere during a year, is a vital process of global water cycles and energy cycles. Revealing the differences in AET values and spatial variations between forests and grasslands would benefit for understanding AET spatial variations, which serves as a basis for regional water management. Based on published eddy covariance measurements in China, we collected AET values from 29 forests and 46 grasslands, and analyzed the differences in AET values and spatial variations between forests and grasslands in China. The results showed that forests had a significant higher AET (645.98 ± 232.73 kgH_2_O m^-2^ yr^-1^) than grasslands (359.31 ± 156.02 kgH_2_O m^-2^ yr^-1^), while the difference in AET values between forests and grasslands was not significant after controlling mean annual precipitation (MAP) relating factors. The effects of latitude and mean annual air temperature (MAT) on AET spatial variations differed between forests and grassland, while AET of forests and grasslands both exhibited increasing trends with similar rates along the increasing MAP, aridity index (AI), soil water content (SW), and leaf area index. The comprehensive effects of multiple factors on AET spatial variations differed between forests and grasslands, while MAP both played a dominating role. The effects of other factors were achieved through their close correlations with MAP. Therefore, forests and grasslands under similar climate had comparable AET values. AET responses to MAP were comparable between ecosystem types. Our findings provided a data basis for understanding AET spatial variation over terrestrial ecosystems of China or globally.

## Introduction

Evapotranspiration (ET), the water loss to the atmosphere as water vapor, is a vital process of global water cycles and energy cycles (Wang and Dickinson, 2012; Douville et al., 2013), which also serves as a key parameter in hydrology and ecology (Wang and Dickinson, 2012; Zhu et al., 2015). Annual evapotranspiration (AET) is the accumulated evapotranspiration during a year. AET spatial variation, resulted from the adaption of an ecosystem to the local environment, serves as the basis for regional water management (Wang et al., 2013; Kool et al., 2014). Revealing AET spatial variation and its affecting factors would benefit for the reasonable utilization of limited regional water resources (Li et al., 2018; Sun et al., 2021).

Based on network eddy covariance measurements (Baldocchi, 2008; Baldocchi, 2014), many works have analyzed AET spatial variations (Brümmer et al., 2012; Krishnan et al., 2012; Xiao et al., 2013; Zheng et al., 2016; Yue et al., 2022). Results found that AET showed a significant declining latitudinal pattern in China (Zheng et al., 2016) resulting from the joint effects of climatic and biological factors (Brümmer et al., 2012; Krishnan et al., 2012; Xiao et al., 2013; Zheng et al., 2016; Yue et al., 2022). However, most previous works investigated the AET spatial variation with all kinds of ecosystem types and ignored the differences in the effects of ecosystem types on AET spatial variations (Brümmer et al., 2012; Xiao et al., 2013; Zheng et al., 2016; Yue et al., 2022), which inhibits our fully understanding of AET spatial variations as different ecosystem types showed divergent environmental statuses. For example, wetlands and some croplands suffered from external water sources (Ding et al., 2010; Zhao et al., 2010), while forests and grasslands seldom had external water supplement.

As the main components of global terrestrial ecosystems, forests and grasslands accounted for more than half of the land surface (O’Mara, 2012; Chen et al., 2020; Hill and Guerschman, 2020; Guo et al., 2022). Revealing the differences in AET spatial variations between forests and grasslands would thus benefit for understanding the AET spatial variations over terrestrial ecosystems. In addition, Chinese forests and grasslands played an important role in global forests and grasslands, respectively (Miao et al., 2013). Furthermore, China experiences a unique climate as the comprehensive effects of Asian monsoon and the uplift of the Qinghai-Tibet Plateau (Wu et al., 2007). Therefore, revealing the differences in AET spatial variations between forests and grasslands in China will help to improve our understanding of AET spatial variations. The widely conducting eddy covariance measurements in divergent ecosystems of China (Yu et al., 2006; Yu et al., 2013; Yu et al., 2016; Zhu et al., 2022; Zhu et al., 2023) accumulated a great deal of AET values thus provided a solid basis for analyzing AET spatial variations, which made it possible to illustrate the differences in AET spatial variations between forests and grasslands.

Therefore, based on eddy covariance measuring AET of forests and grasslands in China, we analyzed the differences in AET values and spatial variations between forests and grasslands to clarify: 1) the differences in AET values between forests and grasslands, 2) the differences in AET spatial variations between forests and grasslands, and 3) the main drivers of the differences in AET values and spatial variations. Our results will improve our understanding on the spatial variation of AET, which also provides a data basis for regional water balance assessment.

## Materials and methods

### Acquisition of measured AET

**Figure 1:**
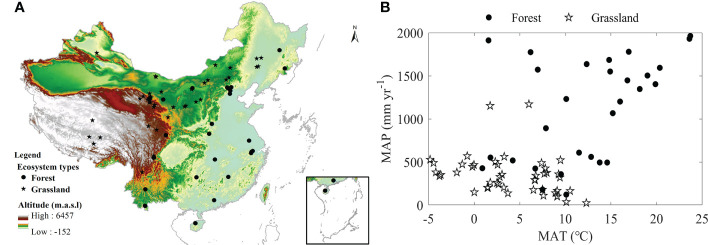
Spatial distribution **(A)** and Climate distribution **(B)** of ecosystems used in this study.

In this study, all measured AET sourced from the published literatures. Using “eddy covariance” and “grassland” or “forest” as the keyword, we searched the published works during 2000–2021 through the core collection of Web of Science (www.isiknowledge.com) and China National Knowledge Infrastructure (www.cnki.net). Each searched result was thoroughly read to extract *in-situ* AET measurements. As we focused on the spatial variations of AET, only measurements with more than 1 year observing AET available were collected. In addition, if an ecosystem had more than 1 year measurements, the mean value of multiyear measurements was calculated to represent its AET value (Yu et al., 2013; Zheng et al., 2016). When collecting AET values, their ecosystem types were simultaneously recorded. The ecosystem types were classified into forests and grasslands, where shrub and desert ecosystems were classified into grasslands. Based on the published data, an AET dataset was constructed containing 75 ecosystems, including 29 forests and 46 grasslands (**Figure 1**). The detailed information of each ecosystem was listed in **Supplementary Table S1**.

### Acquisition of auxiliary data

When collecting measured AET values from literatures, we simultaneously gathered other information, such as the geographical information (latitude, longitude, and altitude) and climatic factors (mean annual air temperature (MAT) and mean annual precipitation (MAP)). However, some ecosystems missed reporting climatic factors, which made the climatic factors incomplete. We extracted the missed climatic factors with geographical information of each ecosystem from their corresponding grid products (Zhu et al., 2022; Zhu et al., 2023), which downscaled from time series data of Climatic Research Unit (CRU) (Harris et al., 2020) to the 30 arc *sec* (~ 1 km) with the delta spatial downscaling (Peng et al., 2019).

Besides MAT and MAP, we also employed other climatic factors like aridity index (AI), annual mean vapor pressure deficit (VPD), annual photosynthetic active radiation (PAR), annual mean CO_2_ mass concentration (*ρ*_c,yr_) to illustrate their effects on AET spatial variations and their differences between ecosystem types, while those climatic factors were seldom reported in literatures. Therefore, we also extracted those factors from their corresponding grid products with the geographical information of each ecosystem. AI was calculated as the ratio of MAP to annual potential evapotranspiration (PET), where PET sourced from the downscaled CRU time series data (Zhu et al., 2022; Zhu et al., 2023). VPD was calculated from MAT and water vapor pressure, which was also downscaled from CRU time series data (Zhu et al., 2022; Zhu et al., 2023). PAR was summed from the daily PAR extracted from Global Land Surface Satellite (GLASS) data at a spatial resolution of 0.05° (Liang et al., 2013; Cheng and Liang, 2014). *ρ*_c,yr_ was calculated from the CO_2_ concentration (*b_c_*), CO_2_ molar mass (*M_c_*, 44 g mol^−1^), and gas molar volume at its current state(*V*), where *b_c_* was replaced by the observed value of Mauna Roa and *V* was calculated by the ideal gas state equation combining local pressure and MAT (Zhu et al., 2016b).

Besides climatic factors, we also employed other factors like soil variables and leaf area index, which were all extracted from their corresponding grid products with geographical information of each ecosystem. Soil variables included annual mean soil moisture (SM), soil organic carbon content (SOC), and soil total nitrogen content (STN). SM was extracted from the soil moisture grid at a spatial resolution of 30 arc *sec* (~ 1 km) downscaled from the remote sensing retrieving data at a spatial resolution of 0.1° and a temporal resolution of 10 day (Chen et al., 2021). SOC and STN were both extracted from a global soil dataset used for earth system models with a spatial resolution of 30 arc *sec* (~ 1 km) (Shangguan et al., 2014). Leaf area index included mean annual leaf area index (LAI) and the maximum leaf area index (MLAI), which were both extracted from an improved Moderate Resolution Imaging Spectroradiometer (MODIS) product with a spatial resolution of 500 m and a temporal resolution of 8 days (Yuan et al., 2011) (http://globalchange.bnu.edu.cn/research/lai).

### Data analysis

Our analysis focused on the differences in AET values and spatial variations between forests and grasslands. The difference in AET values between ecosystem types was conducted with the one-way analysis of variance (ANOVA). Given some factors affected AET values, we employed the analysis of covariance (ANCVA) by fixing some important factors (MAT, MAP, MAP/PET, SW) to reveal the difference in AET values. Before analyzing the difference in AET values, we investigated the difference in environmental factors between forests and grasslands. The differences in AET spatial variations included those in the geographical patterns and the effects of environmental factors on AET spatial variations, which were all conducted with the generalized linear model. In addition, ANCVA was also employed to reveal the difference in AET spatial variations between forests and grasslands.

Considering the ecosystems used in this study covered different measuring period, the interannual variation in AET may introduce some uncertainties by using the mean AET value of measuring period. Therefore, we conducted an uncertainty analysis by randomly adding a within 10% error to the mean values, which was repeated 100 times. The error adding AET were used to analyze AET spatial variations with the generalized linear model. The mean statistics of the 100 repeated regressions were compared to the regression statistics to verify whether AET spatial variations varied with the uncertainties in AET values.

Furthermore, to quantify the comprehensive effects of environmental factors on AET spatial variations and their difference between forests and grasslands, we applied the stepwise analysis to construct the multiple regression equations considering all significant variables. In addition, we employed independence effect analysis to disentangle the relative roles of each factor in AER spatial variations and their difference between forests and grasslands (Murray and Conner, 2009; Chu et al., 2016; Zhu et al., 2022).

### Statistical analysis

In this study, all analyses were conducted using MATLAB software (Math Works Inc., Natick, MA, USA). The differences in environmental factors and AET values were analyzed with ANOVA using the function of “*anova1*”. The difference in AET values was further investigated with ANCVA by fixing main factors as the covariates using the function of “*aoctool*”. The spatial variations of AET were analyzed with the generalized linear model using the function of “*regstats*”. The differences in AET spatial variations were also detected with the ANCVA using the function of “*aoctool*”. The stepwise analysis was employed to reveal the joint effects of environmental factors on AET spatial variations using the function of “*stepwise*”. The minimum P value for introducing into or removing out the regression model was set to 0.10. All significance levels were set to 0.05.

## Results

### Differences in environmental factors and AET values

**Figure 2:**
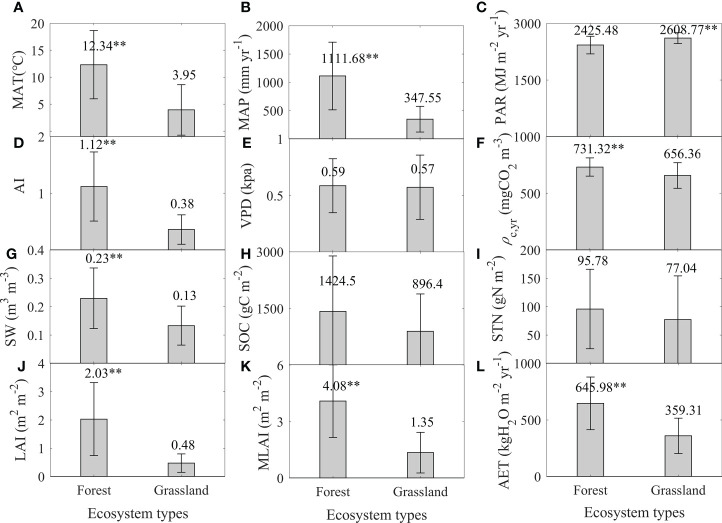
Differences in environmental factors and annual evapotranspiration (AET, **L**) between forests and grasslands. Environmental factors include mean annual air temperature (MAT, **A**), mean annual precipitation (MAP, **B**), annual total photosynthetic effective radiation (PAR, **C**), aridity index (AI, **D**), annual mean water vapor pressure difference (VPD, **E**), annual mean CO_2_ mass concentration (*ρ*_c,yr_, **F**), soil water content (SW, **G**), soil organic carbon content (SOC, **H**), soil total nitrogen content (STN, **I**), mean annual leaf area index (LAI, **J**), and maximum leaf area index (MLAI, **K**). *P<.05, **P<.01, ***P<.001.

Forests and grasslands had divergent environmental factors and AET values, while the significance levels differed among factors (**Figure 2**).

Major climatic factors, including MAT, MAP, and PAR, significantly differed between forests and grasslands, while the differing directions varied among factors (**Figures 2A–C**). Forests had a higher MAT (12.34 ± 6.33 °C) and MAP (1111.68 ± 597.19 mm yr ^-1^) than those of grasslands (F = 43.29 and 61.51, P< 0.01), while the PAR of forests (2425.48 ± 232.99 MJ m^-2^ yr^-1^) was significantly lower than that of grasslands (2608.77 ± 143.03 MJ m^-2^ yr^-1^) (F = 17.86, P< 0.01).

Other climatic factors showed divergent differences between forests and grasslands (**Figures 2D–F**). Forests and grasslands had similar VPD values (**Figure 2E**), while the *ρ*_c,yr_ of forests was significantly lower than that of grasslands (F = 9.67, P< 0.01). In addition, the aridity index (AI), defined as the ratio of MAP to PET, significantly differed between forests and grasslands. Forests took a significant higher AI (1.12 ± 0.61) than grasslands (0.38 ± 0.26) (F = 52.72, P< 0.01).

Soil variables also exhibited divergent differences between forests and grasslands (**Figures 2G–I**). Forests had a significant higher SW (0.23 ± 0.11 m^3^ m^-3^) than grasslands (F = 22.6, P<0.01), while there was no significant difference in SOC and STN between forests and grasslands (P > 0.05).

Forests took higher leaf area index (LAI and MLAI) than grasslands (**Figures 2J, K**). The LAI of forests reached to 2.03 ± 1.29 m^2^ m^-2^, which was significantly higher than that of grasslands (0.48 ± 0.33 m^2^ m^-2^) (F = 61.1, P< 0.01). The difference in MLAI also showed a similar trend (F = 61.9, P< 0.01).

Forests took a significant higher AET than grasslands, whereas their differences were primarily attributed to the differences in the receiving water amount as MAP (**Figure 2L**). Forests took an AET value of (645.98 ± 232.73 kgH_2_O m^-2^ yr^-1^), which was significantly higher than that of grassland (359.31 ± 156.02 kgH_2_O m^-2^ yr^-1^) (F = 40.85, P< 0.01). After fixing the effects of MAP or AI, the differences in AET values between forests and grasslands were not significant (P > 0.05). However, setting MAT or SW as the covariant, forests still had a significant higher AET than grasslands (P< 0.01). Considering the dominating role of MAP in AI, the difference in AET values between forests and grasslands primarily sourced from that in the receiving water amount as MAP.

### Differences in AET geographical patterns

**Figure 3:**
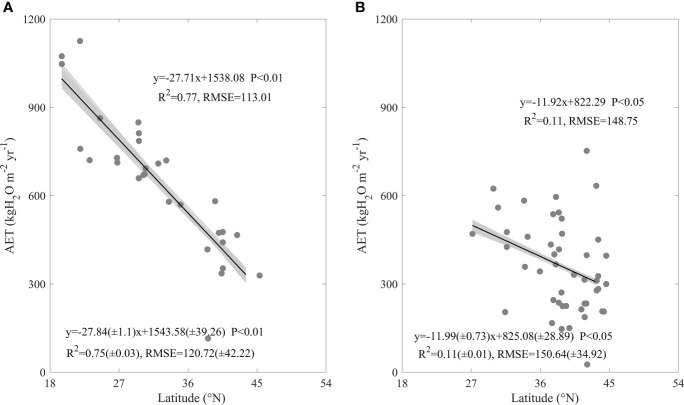
The annual evapotranspiration (AET) latitudinal patterns of forests **(A)** and grasslands **(B)** in China. The grey lines are regression lines generated from the random error adding AET, whose statistics are presented in the left bottom of each panel.

Both forests and grasslands showed significant decreasing latitudinal patterns, while the decreasing rates of AET significantly differed between forests and grasslands (**Figure 3**). With the increasing latitude, forest AET significant decreased, with a decreasing rate of 27.71 kgH_2_O m^-2^ yr^-1^. The equation containing latitude explained 77% AET spatial variation in forest, with an RMSE of 113.01 kgH_2_O m^-2^ yr^-1^ (**Figure 3A**). The increasing latitude also significantly decreased AET in grasslands, while its decreasing rate was only 11.92 kgH_2_O m^-2^ yr^-1^, with an R^2^ of 0.11 and an RMSE of 148.75 kgH_2_O m^-2^ yr^-1^ (**Figure 3B**). Forests took a significant higher decreasing rate than grasslands (F = 7.37, P< 0.01). In addition, the decreasing latitudinal patterns of AET and their difference between forests and grasslands did not vary with the uncertainties in AET, indicated by the similar regression statistics from the error adding AET (**Figure 3**).

In contrast to the significant decreasing latitudinal patterns, AET of forests and grasslands both did not exhibit significant longitudinal and altitudinal patterns (data not shown).

### Differences in the effects of environmental factors on AET spatial variations

Divergent environmental factors exerted different effects on AET spatial variations, only MAT, MAP, AI, SW, LAI, and MLAI exhibited strong effects on AET spatial variations, while the differences in their effects between forests and grasslands varied among factors.

**Figure 4:**
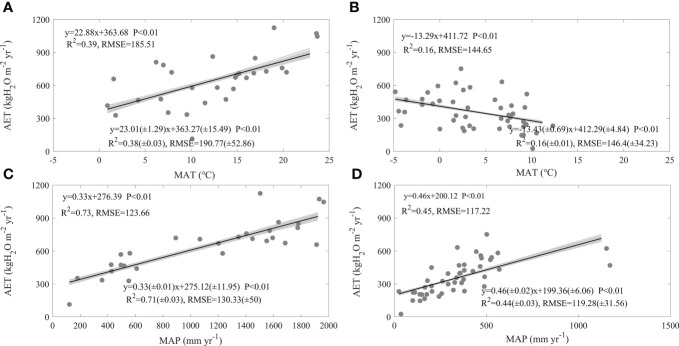
Effects of mean annual air temperature (MAT, **A, B**) and mean precipitation (MAP, **C, D**) on the spatial variations of annual evapotranspiration (AET) in forests **(A, C)** and grasslands **(B, D)** in China. The grey lines are regression lines generated from the random error adding AET, whose statistics are presented in the right bottom of each panel.

MAT significantly affected the spatial variations of AET both in forests and grasslands, while its effects differed in directions between forests and grasslands (**Figures 4A, B**). With increasing MAT, AET of forests showed a significant increasing trend at a rate of 22.88 kgH_2_O m^-2^ yr^-1^, with an R^2^ of 0.39 and an RMSE of 185.51 kgH_2_O m^-2^ yr^-1^ (**Figure 4A**). However, the increasing MAT significantly decreased AET in grasslands. Each increase in MAT decreased the grassland AET by 13.29 kgH_2_O m^-2^ yr^-1^. The equation containing MAT explained 16% of the spatial variation in grassland AET, with an RMSE of 144.65 kgH_2_O m^-2^ yr^-1^ (**Figure 4B**). The effects of MAT on AET spatial variations significantly differed between forests and grasslands (F = 26.41, P< 0.01). The effects of MAT and their differences between ecosystem types varied little with the error adding AET (**Figures 4A, B**).

MAP exerted similar promotions on AET spatial variations both in forests and grasslands (**Figures 4C, D**). The increasing MAP significantly increased forest AET at a rate of 0.33 kgH_2_O m^-2^ yr^-1^, with an R^2^ of 0.73 and an RMSE of 123.66 kgH_2_O m^-2^ yr^-1^ (**Figure 4C**). The increasing MAP raised grassland AET at a rate of 0.46 kgH_2_O m^-2^ yr^-1^. The equation containing MAP explained 45% AET spatial variation in grasslands, with an RMSE of 117.22 kgH_2_O m^-2^ yr^-1^ (**Figure 4D**). The increasing rates of AET induced by MAP showed no significant difference between forests and grasslands (F = 2.09, P > 0.05). In addition, the effects of MAP on AET spatial variations and their differences between ecosystem types did not vary with the error adding AET (**Figures 4C, D**).

**Figure 5:**
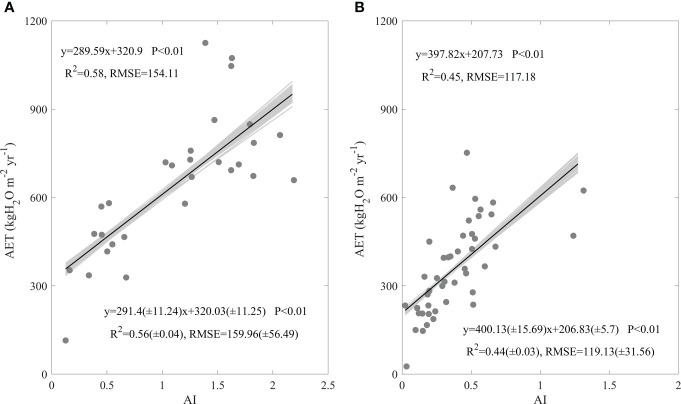
Effects of aridity index **(AI)** on the spatial variations of annual evapotranspiration (AET) in forests **(A)** and grasslands **(B)** in China. The grey lines are regression lines generated from the random error adding AET, whose statistics are presented in the right bottom of each panel.

AI also exerted significant positive effects on AET spatial variations both in forests (**Figure 5A**) and grasslands (**Figure 5B**), with no significant difference appearing between ecosystem types (**Figure 5**). The increasing AI made forest AET significantly increase at a rate of 289.59 kgH_2_O m^-2^ yr^-1^, with an R^2^ of 0.58 and an RMSE of 154.11 kgH_2_O m^-2^ yr^-1^ (**Figure 5A**). Grassland AET was also raised by the increasing AI at a rate of 397.82 kgH_2_O m^-2^ yr^-1^, with an R^2^ of 0.45 and an RMSE of 117.18 kgH_2_O m^-2^ yr^-1^(**Figure 5B**). Though the increasing rates differed in values between forests and grasslands, their difference was not statistically different (F = -1.26, P > 0.05) (**Figure 5**). In addition, the errors in AET calculation indicated by the error adding AET seldom varied the effects of AI on AET spatial variations and their differences between ecosystem types (**Figure 5**).

**Figure 6:**
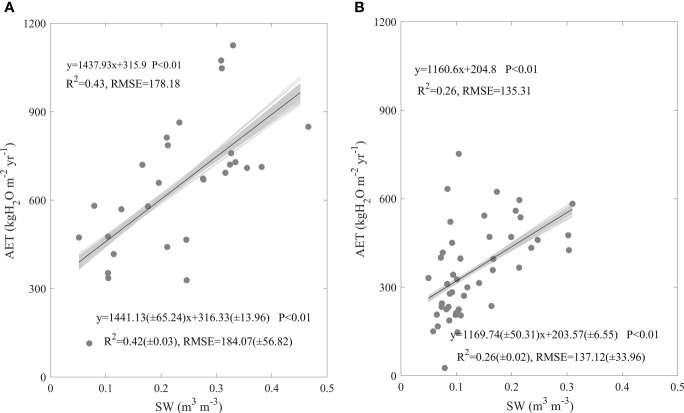
Effects of soil water content (SW) on the spatial variations of annual evapotranspiration (AET) in forests **(A)** and grasslands **(B)** in China. The grey lines are regression lines generated from the random error adding AET, whose statistics are presented in the right bottom of each panel.

The increasing SW significantly increased AET in forests and grasslands at similar rates (**Figure 6**). With the increasing SW, Forest AET significantly increased at a rate of 1437.93 kgH_2_O m^-2^ yr^-1^. The equation containing SW explained 43% of AET spatial variation in forests, with an RMSE of 178.18 kgH_2_O m^-2^ yr^-1^ (**Figure 6A**). Grassland AET increased with the increasing SW at a rate of 1160.6 kgH_2_O m^-2^ yr^-1^, with an R^2^ of 0.26 and an RMSE of 135.31 kgH_2_O m^-2^ yr^-1^(**Figure 6B**). The increasing rates of AET along the increasing SW showed no significant difference between forests and grasslands (F = 0.42, P > 0.05). Meanwhile, the potential errors in calculating AET seldom varied the effects of SW on AET spatial variations and their difference between forests and grasslands (**Figure 6**).

**Figure 7:**
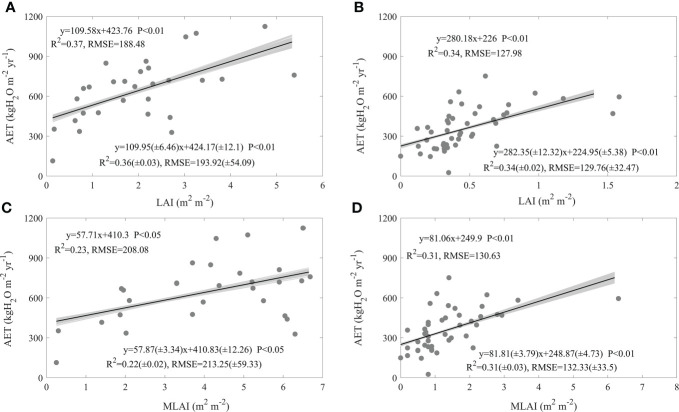
Effects of mean annual leaf area index (LAI, **A, B**) and maximum leaf area index (MLAI, **C, D**) on the spatial variations of annual evapotranspiration (AET) in forests **(A, C)** and grasslands **(B, D)** in China. The grey lines are regression lines generated from the random error adding AET, whose statistics are presented in the right bottom of each panel.

Leaf area index exerted significant and positive effects on AET spatial variations, but their effects differed between forests and grasslands (**Figure 7**). The increasing LAI made forest AET increase at a rate of 109.58 kgH_2_O m^-2^ yr^-1^, with an R^2^ of 0.37and an RMSE of 188.48 kgH_2_O m^-2^ yr^-1^ (**Figure 7A**). The increasing LAI also made grassland AET increase, while the increasing rate was 280.18 kgH_2_O m^-2^ yr^-1^, with an R^2^ of 0.34 and an RMSE of 127.98 kgH_2_O m-^2^ yr^-1^ (**Figure 7B**). Forests took a significant higher AET increasing rate than grasslands (F = 5.33, P< 0.05). With increasing MLAI, forest AET increased at a rate of 57.71 kgH_2_O m^-2^ yr^-1^, with an R^2^ of 0.23 and an RMSE of 208.08 kgH_2_O m^-2^ yr^-1^ (**Figure 7C**), while grassland AET increased at a rate of 81.06 kgH_2_O m^-2^ yr^-1^, with an R^2^ of 0.31 and an RMSE of 130.63 kgH_2_O m^-2^ yr^-1^ (**Figure 7D**). Though the increasing rates along the increasing MLAI differed in values between forests and grasslands, their difference was not statistically significant (F = 0.7, P > 0.05). In addition, the uncertainties in calculating AET seldom affected the effects of leaf area index on AET spatial variations and their differences between forests and grasslands (**Figure 7**).

Based on the unique effects of each factors, we obtained their comprehensive effects on AET spatial variations in forests and grasslands and their difference between ecosystem types. Results showed that climatic factors dominated the spatial variation of AET in forests, with the dominating role of MAP. The equation containing MAP, AI, and *ρ*_c,yr_ explained 83% of AET spatial variation in forests, with an RMSE of 104.40 kgH_2_O m^-2^ yr^-1^ (Eq. (1)). MAP had a higher independent effect accounting for 48% of AET spatial variation. AI accounted for 33% of AET spatial variation, while the independent effect of *ρ*_c,yr_ was only 2%. However, the spatial variation of AET was jointly affected by MAP and leaf area index with the dominating role of MAP. The equation containing MAP, LAI, and MLAI explained 56% of AET spatial variation in forests, with an RMSE of 106.87 kgH_2_O m^-2^ yr^-1^ (Eq. (2)). MAP had a higher independent effect accounting for 28% of AET spatial variation, while LAI and MLAI both accounted for 14% of AET spatial variation. Therefore, MAP exerted a stronger effect on AET spatial variations both in forests and grasslands.


(1) $$\text{AET}=0.76\text{MAP}-423.75\text{AI}-0.50\rho_{\text{c},\text{yr}}+645.73,{\text{ R}}^{2}=0.83,\text{ RESE}=103.07,\text{ n}=29,\text{P}<0.01$$

(2) $$\text{AET}=0.49\text{MAP}-324.39\text{LAI}+123.84\text{MLAI}+176.80,{\text{ R}}^{2}=0.56,\text{ RESE}=106.87,\text{ n}=45,\text{ P}<0.01$$

## Discussions

### The differences in AET values

In this study, we found that AET values significantly differed between forests and grasslands (**Figure 2L**), while the difference in AET values was not significant after controlling MAP relating factors as the covariant. Our results indicate that the differences in AET values between forests and grasslands may primarily source from the difference in MAP, which mainly resulted from the spatial distribution of forests and grasslands used in this study. Forests employed in this study primarily distributed in eastern China, while grasslands were mainly located in the western China (**Figure 1**) (Guo et al., 2022), which made forests have a higher MAP than grasslands (**Figure 2B**). A higher MAP meant more water were available for an ecosystem to evaporate as the energy arriving at the land surface were much larger than that an ecosystem required for evaporation (Jin et al., 2011; Chapin et al., 2012; Williams et al., 2012; Zhu et al., 2016a; IPCC, 2021). Under similar MAP, both forests and grasslands had similar amounts of available water for evaporating, which made AET values of forests and grasslands comparable. Therefore, the spatial distribution of forests and grasslands induced the difference in MAP, which shaped the significant difference in AET values between forests and grasslands. The fact that forests had a higher AET than grasslands was widely found in direct measurements (Brümmer et al., 2012; Xiao et al., 2013; Zheng et al., 2016; Feltrin et al., 2017) or modeling results (Li et al., 2014; Li et al., 2018; Fang et al., 2020; Sun et al., 2021), while our results originally found that forests and grasslands had similar AET values after controlling MAP relating factors as the covariant. This phenomenon meant that forests and grasslands had comparable AET values under similar MAP, which may indicate that different ecosystem types under similar climate had comparable AET values. Therefore, MAP gradient may be more important than ecosystem types in determining AET values, which meant that selecting parameters for modeling ET thus AET may take MAP as a reference but not only ecosystem types.

### Differences in AET spatial variations

In this study, we found factors shaping AET spatial variations differed between forests and grasslands, while MAP played a dominating role both in forests and grasslands (Eqs. (1-2)). In addition, the AET increasing rates along the increasing MAP showed no significant difference between ecosystem types (**Figures 4C, D**). AI (**Figure 5**), SW (**Figure 6**), and leaf area index (**Figure 7**) all exhibited similar effects with MAP. However, MAT exerted divergent effects on AET spatial variations between forests and grasslands (**Figures 4A, B**), so did latitude (**Figure 3**). As an annual value at a relative long time scale, AET reflected the comprehensive adaption of an ecosystem to local environment, which compromised between water supply and energy demand. MAP directly provided the available water for evaporation (Chapin et al., 2012; Wang and Dickinson, 2012). Considering the available energy used for evapotranspiration represented by net radiation was much higher than that evapotranspiration required (Jin et al., 2011; Chapin et al., 2012; Williams et al., 2012; Zhu et al., 2016a; IPCC, 2021), MAP played a dominating role in AET spatial variations. However, the post processes after water arriving at the land surface like runoff or infiltration and vegetation metabolism limited the representativeness of MAP as water supply, which made AET not respond to MAP in a direct linear way (**Figures 4C, D**) (Williams et al., 2012). The similar effects of AI, SW, and leaf area index on AET spatial variations with MAP may source from their close correlation with MAP (**Supplementary Table S2**). MAP showed high correlation coefficients with those factors, which were found both in forests and grasslands (**Supplementary Table S2**). In addition, MAP showed divergent correlation coefficients with MAT between forests and grasslands (**Supplementary Table S2**) as the site spatial distribution (**Figure 1**), which induced the divergent effects of MAT on AET spatial variations between forests and grasslands (**Figures 4A, B**). The negative correlation coefficients between latitude and MAP made AET show significant decreasing latitudinal pattern (**Figure 3**). However, the MAP decreasing rates along the increasing latitude significantly differed between forests and grasslands (F = -3.93, P<0.01, data were not shown), which induced the significant difference in AET latitudinal patterns between forests and grasslands (**Figure 3**). Therefore, it was well known that MAP affected the spatial variation of AET, while we originally revealed the dominating role of MAP in AET spatial variations. In addition, we originally found the similar increasing rates of AET along the increasing MAP between forests and grasslands, which indicates that AET of different ecosystem types responded similar to MAP.

## Conclusions

Based on published eddy covariance measurements in China, we collected annual evapotranspiration (AET) data from 29 forests and 46 grasslands, and analyzed the differences in AET values and spatial variations between forests and grasslands in China. Results showed that forests had a higher AET than grasslands, while similar AET values occurred between forests and grasslands after controlling mean annual precipitation (MAP) relating factors as the covariant. Factors shaping AET spatial variations differed between forests and grasslands, while MAP played a dominating role both in forests and grasslands. Along the increasing MAP, AET increased at the similar rates between forests and grasslands. Therefore, different ecosystem types under similar climate had comparable AET values. AET responses to MAP were comparable between ecosystem types. Our findings provided a data basis for understanding AET spatial variation over terrestrial ecosystems of China or globally.

## Data availability statement

The original contributions presented in the study are included in the article/**Supplementary material**. Further inquiries can be directed to the corresponding authors.

## Author contributions

RF, analysis of data and drafts the manuscript; MS, acquisition of data and designs MATLAB program; XZ, propose the design of the manuscript and makes suggestions for revision; QW, approves the final manuscript. All authors contributed to the article and approved the submitted version.

## Funding

The work was jointly supported by the CAS Strategic Priority Research Program (XDA19020302), the National Natural Science Foundation of China (32071585, 32071586, and 31500390), and Special Foundation for National Science and Technology Basic Research Program of China (2019FY101303-2).

## Acknowledgments

We gratefully acknowledge the reviewers for spending their valuable time to provide constructive comments. Special thanks to data provider from the FLUXNET community especially ChinaFLUX and USCCC networks.

## Conflict of interest

The authors declare that the research was conducted in the absence of any commercial or financial relationships that could be construed as a potential conflict of interest.

## Publisher’s note

All claims expressed in this article are solely those of the authors and do not necessarily represent those of their affiliated organizations, or those of the publisher, the editors and the reviewers. Any product that may be evaluated in this article, or claim that may be made by its manufacturer, is not guaranteed or endorsed by the publisher.
