# Supplementary material for: Eddy covariance measurement-based differences in annual evapotranspiration between forests and grasslands in China
Source: Front Plant Sci. 2022 Nov 25;13:1030929. doi: 10.3389/fpls.2022.1030929 (PMC9732459; doi:10.3389/fpls.2022.1030929)
Supplement: Supplementary file 1 [file DataSheet_1.docx]

Supplementary Material

Supplementary Table S1 Brief information of sites used in this study

| No. | Site Name | Latitude (°N) | Longitude (°E) | AET (kgH_2_O m^-2^ yr^-1^) | Measuring period | References |
| --- | --- | --- | --- | --- | --- | --- |
| **Forests** | | | | | | |
| 1 | Danzhou Rubber2 | 19.54 | 109.47 | 127 | 2013-2019 | ChinaFLUX |
| 2 | Danzhou Rubber1 | 19.55 | 109.48 | 132 | 2017-2019 | Geng et al., 2020 |
| 3 | Xishuangbanna Rubber | 21.93 | 101.27 | 552 | 2011 | Song et al., 2014 |
| 4 | Xishuangbanna Forest | 21.96 | 101.21 | 758 | 2003-2008 | Zhang et al., 2010 |
| 5 | Dinghushan Forest | 23.17 | 112.54 | 380 | 2003-2011 | Yan et al., 2013  liu et al., 2015 |
| 6 | Ailaoshan Forest | 24.54 | 101.03 | 2508 | 2009-2016 | Tan et al., 2011  Song et al., 2017 |
| 7 | Qianyanzhou Forest | 26.74 | 115.06 | 112 | 2003-2014 | Xu et al., 2017 |
| 8 | Huitong forest | 26.79 | 109.59 | 313 | 2008 | Zhao et al., 2011 |
| 9 | Yueyang forest | 29.53 | 112.86 | 28 | 2010-2013 | Gao et al., 2015 |
| 10 | Gonggashan Forest1 | 29.57 | 101.99 | 3808 | 2015 | Zhang et al., 2018 |
| 11 | Gongga mountain mixed forest | 29.59 | 102.03 | 2976 | 2016 | Sun et al., 2020 |
| 12 | Gonggashan Forest2 | 29.6 | 102.05 | 2953 | 2016 | Sun et al., 2020 |
| 13 | Linan forest | 30.18 | 119.34 | 113 | 2011-2016 | Liu et al., 2018 |
| 14 | Tianmushan Forest | 30.35 | 119.44 | 1229 | 2013-2014 | Niu et al., 2016  Fang, 2016 |
| 15 | Anji Forest | 30.48 | 119.67 | 282 | 2011-2016 | Liu, 2018  Song et al., 2017 |
| 16 | Jurong Forest | 32.12 | 119.2 | 219 | 2008, 2011 | Zhang, 2010  Li, 2012 |
| 17 | Jiuzhaigou Forest | 33.16 | 103.88 | 3029 | 2014-2015 | Yan et al., 2017 |
| 18 | Baotianman Forest | 33.5 | 111.94 | 1328 | 2017 | Niu et al., 2018 |
| 19 | Xiaolangdi forest | 35.02 | 112.47 | 353 | 2008-2010 | Tong et al., 2017 |
| 20 | Guantan Forest | 38.53 | 100.25 | 2868 | 2011 | Zhu et al., 2014 |
| 21 | Minqin | 38.6 | 102.95 | 1380 | 2014-2015 | Zhang et al., 2016 |
| 22 | Daxing Forest | 39.53 | 116.25 | 36 | 2007-2009 | Kang et al., 2015 |
| 23 | Aolinpike park forest | 40.02 | 116.38 | 41 | 2012-2014 | Xie et al., 2016 |
| 24 | Badaling Forest | 40.37 | 115.94 | 525 | 2012-2017 | Ma et al., 2018  Ma et al., 2019 |
| 25 | Songshan forest | 40.51 | 115.79 | 944 | 2019 | Li et al., 2020 |
| 26 | Miyun Forest | 40.53 | 116.62 | 1007 | 2015 | Tie et al., 2018 |
| 27 | Kubuqi Forest | 40.54 | 108.69 | 1027 | 2005-2006 | FLUXNET2015 |
| 28 | Changbaishan Forest | 42.4 | 128.1 | 751 | 2003-2019 | Zhang et al., 2012 ChinaFLUX |
| 29 | Laoshan Forest | 45.33 | 127.67 | 672 | 2004, 2008 | Wang et al., 2008  Qiu et al., 2011 |
| **Graassland** | | | | | | |
| 30 | Lijiang Grassland | 27.17 | 100.23 | 3618 | 2012-2015 | Wang et al., 2017 |
| 31 | Gongga Mountain shrubland | 29.89 | 102.01 | 3968 | 2016 | Sun et al., 2020 |
| 32 | Dangxiong Grassland | 30.5 | 91.07 | 4367 | 2004-2008 | Chai et al., 2018 |
| 33 | Bange Grassland | 31.42 | 90.03 | 4719 | 2015 | Wang et al., 2018 |
| 34 | Naqu Fenced Grassland | 31.64 | 92.01 | 4609 | 2012-2019 | Zhang et al., 2018  ChinaFLUX |
| 35 | Naqu Grassland | 31.65 | 92 | 4619 | 2012-2014 | Zhang et al., 2015  An et al., 2019 |
| 36 | Ruoergai Grassland | 33.89 | 102.14 | 3433 | 2010-2011 | Shang et al., 2016 |
| 37 | Shuanghu Grassland | 34 | 90 | 4988 | 2012-2013 | Ma et al., 2015 |
| 38 | Sanjiangyuan Grassland | 34.35 | 100.55 | 4076 | 2006, 2008 | Tian et al., 2020 |
| 39 | Lanzhou Grassland | 35.95 | 104.13 | 1778 | 2007-2013 | Ping et al., 2018  Yang et al., 2019 |
| 40 | Haibei Grassland | 37.37 | 101.18 | 3823 | 2015-2016 | Li et al., 2018  Kato et al., 2006 |
| 41 | Shapotou Grassland | 37.53 | 105.03 | 1304 | 2009-2012 | Gao et al., 2012  Gao et al., 2016 |
| 42 | Haibei Shrubland | 37.68 | 101.34 | 3422 | 2003-2012 | Li et al., 2016 |
| 43 | Yanchi Shrubland | 37.71 | 107.23 | 1542 | 2012-2016 | Jia et al., 2016  Jia et al., 2020 |
| 44 | Yanchi Desert | 37.83 | 107.49 | 1421 | 2017, 2019 | Wang, 2020 |
| 45 | Yakou Grassland | 38.01 | 100.24 | 4039 | 2015 | Sun et al., 2019  Wang et al., 2019 |
| 46 | Arou Grassland | 38.05 | 100.46 | 2985 | 2013-2019 | Zhang et al., 2020  ChinaFLUX |
| 47 | Shule Grassland | 38.42 | 98.32 | 3875 | 2008-2012 | Wu et al., 2020 |
| 48 | Sunan Grassland | 38.42 | 99.86 | 4043 | 2013-2018 | Gao, 2020 |
| 49 | Yulin Grassland | 38.45 | 109.47 | 1235 | 2012-2015 | Gong et al., 2018 |
| 50 | Huazhaizi Desert | 38.77 | 100.32 | 1729 | 2012 | Wang et al., 2019 |
| 51 | Shenshawo Desert | 38.79 | 100.49 | 1544 | 2012 | Wang et al., 2019 |
| 52 | Liudaogou Grassland | 38.79 | 110.37 | 1154 | 2012-2016 | Qi et al., 2019 |
| 53 | Dashalong Grassland | 38.84 | 98.94 | 3797 | 2013-2016 | Wang et al., 2019 |
| 54 | Bajitan Grassland | 38.92 | 100.3 | 1564 | 2012-2014 | Wang et al., 2019 |
| 55 | Linze Grassland | 39.38 | 100.13 | 1384 | 2008-2010 | Zhao et al., 2016 |
| 56 | Badain Jaran Grassland | 39.79 | 102.44 | 1194 | 2012 | Hu et al., 2015 |
| 57 | Kubuqi Grassland | 40.38 | 108.55 | 1173 | 2006 | Gilmanov et al., 2010 |
| 58 | Xilamuren Grassland | 41.36 | 111.17 | 1651 | 2013 | Zhang et al., 2016 |
| 59 | Guyuan Grassland | 41.77 | 115.68 | 1389 | 2012-2013 | Xin et al., 2014 |
| 60 | Siziwang Grassland1 | 41.78 | 111.9 | 1432 | 2010-2011 | Shao et al., 2017 |
| 61 | Siziwang Grassland2 | 41.79 | 111.9 | 1430 | 2010-2012 | FLUXNET2015 |
| 62 | Luodi | 42 | 101.13 | 936 | 2012-2014 | Wang et al., 2019 |
| 63 | Duolun Grassland | 42.05 | 116.28 | 1313 | 2005-2008 | ChinaFLUX |
| 64 | Duolun Degraded Grassland | 42.06 | 116.28 | 1317 | 2009-2010 | FLUXNET2015 |
| 65 | Desert | 42.11 | 100.99 | 924 | 2015-2016 | Wang et al., 2019 |
| 66 | Horqin Grassland | 43.29 | 122.28 | 201 | 2008-2013 | Li et al., 2016 |
| 67 | Tongliao Grassland | 43.35 | 122.65 | 205 | 2015 | Gong et al., 2018 |
| 68 | Horqin Deserat | 43.35 | 122.65 | 205 | 2013-2016 | Chen, 2018 |
| 69 | Neimeng Grassland | 43.55 | 116.68 | 1255 | 2004-2014 | ChinaFLUX |
| 70 | Xilinhaot fenced Grassland | 43.55 | 116.67 | 1246 | 2006 | Chen et al., 2009 |
| 71 | Xilinhot Degraded Grassland | 43.55 | 116.67 | 1246 | 2006 | Chen et al., 2009 |
| 72 | Xilinguole Grassland | 44.08 | 113.57 | 971 | 2008-2010 | Wang et al., 2018 |
| 73 | Fukang Grassland | 44.28 | 87.93 | 475 | 2004-209 | Liu et al., 2016 |
| 74 | Tongyu Grassland | 44.59 | 122.52 | 169 | 2011-2017 | Zhao et al., 2019 |
| 75 | Changling Grassland | 44.59 | 123.51 | 141 | 2009-2020 | Qu et al., 2016  ChinaFLUX |

Supplementary Table S2 Correlation coefficients between environment factors in Forests(F) and Grasslands(G)

| F  G | LAT | MAT | MAP | PAR | AI | VPD | *ρ*_c,yr_ | SW | SOC | STN | LAI | MLAI |
| --- | --- | --- | --- | --- | --- | --- | --- | --- | --- | --- | --- | --- |
| LAT |  | -0.72 | -0.87 | -0.26 | -0.76 | 0.06 | 0.06 | -0.55 | 0.08 | 0.28 | -0.61 | -0.35 |
| MAT | 0.29 |  | 0.48 | 0.53 | 0.30 | 0.49 | 0.19 | 0.61 | -0.56 | -0.58 | 0.50 | 0.23 |
| MAP | -0.58 | -0.32 |  | -0.13 | 0.97 | -0.38 | 0.08 | 0.48 | 0.14 | -0.07 | 0.49 | 0.43 |
| PAR | -0.05 | 0.32 | -0.64 |  | -0.33 | 0.72 | 0.11 | 0.36 | -0.68 | -0.51 | 0.23 | -0.18 |
| AI | -0.65 | -0.41 | 0.98 | -0.61 |  | -0.53 | 0.05 | 0.32 | 0.29 | 0.02 | 0.39 | 0.40 |
| VPD | 0.53 | 0.86 | -0.65 | 0.51 | -0.73 |  | 0.21 | 0.20 | -0.74 | -0.64 | 0.02 | -0.24 |
| *ρ*_c,yr_ | 0.63 | 0.51 | -0.35 | 0.02 | -0.43 | 0.58 |  | 0.20 | -0.63 | -0.40 | -0.18 | -0.19 |
| SW | -0.33 | -0.31 | 0.01 | 0.12 | 0.06 | -0.22 | -0.38 |  | -0.24 | -0.21 | 0.28 | 0.15 |
| SOC | -0.63 | -0.51 | 0.71 | -0.44 | 0.76 | -0.65 | -0.64 | 0.38 |  | 0.78 | -0.07 | 0.16 |
| STN | -0.62 | -0.52 | 0.72 | -0.44 | 0.76 | -0.66 | -0.65 | 0.33 | 0.99 |  | -0.11 | 0.11 |
| LAI | -0.40 | -0.22 | 0.65 | -0.36 | 0.64 | -0.45 | -0.39 | 0.19 | 0.60 | 0.63 |  | 0.86 |
| MLAI | -0.27 | -0.32 | 0.47 | -0.32 | 0.48 | -0.45 | -0.41 | 0.26 | 0.50 | 0.53 | 0.93 |  |

LAT: latitude; MAT: mean annual air temperature; MAP: mean annual precipitation; PAR: annual total photosynthetic effective radiation; AI: aridity index; VPD: saturated water vapor pressure difference; *ρ*_c,yr_: annual mass CO_2_ concentration; SW: soil water content; SOC: soil organic carbon content; STN: soil total nitrogen content; LAI: mean annual leaf area index; MLAI: maximum leaf area index.

**References**

An, T., Xu, M., Zhang, T., Yu, C., Li, Y., Chen, N., et al. (2019). Grazing alters environmental control mechanisms of evapotranspiration in an alpine meadow of the Tibetan Plateau. *J. Plant Ecol*. 12(5), 834-845. [doi:10.1093/jpe/rtz021](https://doi.org/10.1093/jpe/rtz021).

Chai, X., Li, Y., Duan, C., Zhang, T., Zong, N., Shi, P., et al. (2018). CO_2_ flux dynamics and its limiting factors in the alpine shrub-meadow and steppe-meadow on the Qinghai-Xizang Plateau. *Chin. J. Plant Ecol*. 42(1), 6-19. doi: 10.17521/ cjpe. 20 17.0266.

Chen, S., Chen, J., Lin, G., Zhang, W., Miao, H., Wei, L., et al. (2009). Energy balance and partition in Inner Mongolia steppe ecosystems with different land use types. *Agr. Forest Meteorol*. 149(11), 1800-1809. doi:10.1016/ j.agrformet. 2009. 06.009.

Chen, X. (2018). *Variations and Influence Mechanism of Carbon and Water Flux in Horqin Dune and Meadow Wetland Landscape* (Hohhot, Inner Mongolia, China: MS Thesis of Inner Mongolia Agricultural University).

Fang, C., Jiang, H., Niu X., Chen X., and Sun H. (2016). Energy flux and balance analysis of evergreen and deciduous broad-leaved mixed forest in Tianmu Mountain during growing season. *J. Fujian Agri. Forestry Univ*. 1671-5470(2016)04-0391-07, doi:1013323/j.cnki.j.fafu (nat.sci.).2016.0.005.

Gao, S., Chen, J., Tang, Y., Xie, J., Zhang, R., Tang, J., et al. (2015). Ecosystem carbon (CO_2_ and CH_4_) fluxes of a Populus dettoides plantation in subtropical China during and post clear-cutting. *Forest Ecol. Manag*. 357, 206-219. doi: 10.1016/ j.foreco. 2015.08.026.

Gao, Y., Li, X., Liu, L., Jia, R., Yang, H., Li, G., et al. (2012). Seasonal variation of carbon exchange from a revegetation area in a Chinese desert. *Agr. Forest Meteorol*. 156, 134-142. doi: 10.1016/j.agrformet.2012.01.007.

Gao, Y., Liu, L., Jia, R., Yang, H., and Li, G. (2016). Evapotranspiration over artificially planted shrub communities in the shifting sand dune area of the Tengger Desert, north central China. *Ecohydrology* 9(2), 290-299. [doi:10.1002/eco.1635](https://doi.org/10.1002/eco.1635).

Gao, Y. (2020). *Evapotranspiration and water balance of subalpine meadows in the Qilian Mountains* (Lanzhou, Gansu, China: MS Thesis of Lanzhou University). doi: 10.27204/d.cnki.glzhu.2020.000116.

Geng, S., Wu, Z., and Yang, C. (2021). Water Vapor Flux Exchange of Rubber Forest Stand in Hainan Danzhou and Its Response to Environmental Factors. *J. Nw. Forestry Univ*. 36(01):77-85.

Gong, T., Lei, H., Yang, D., Yang, H., Liu, Y., and Duan, L. (2018). Assessing impacts of extreme water and temperature conditions on carbon fluxes in two desert shrublands. *J. Hydroelectric Eng*. 37(2): 32-46.

Gilmanov, T., Aires, L., Barcza, Z., Baron, V., Belelli, L., Beringer, J., et al. (2010). Productivity, respiration, and light-response parameters of world grassland and agroecosystems derived from flux-tower measurements. *Rangeland Ecol. Manag*. 63(1), 16-39. doi:10.2111/REM-D-09-00072.1.

Hu, W., Wang, N., Zhao, L., Ning, K., Zhang, X., and Sun, J. (2015). Surface energy and water vapor fluxes observed on a megadune in the Badain Jaran Desert, China. *J. Arid Land* 7(5), 579-589. doi:10.1007/s40333-015-0129-6.

Jia, X., Mu, Y., Zha, T., Wang, B., Qin, S., and Tian, Y. (2020). Seasonal and interannual variations in ecosystem respiration in relation to temperature, moisture, and productivity in a temperate semi-arid shrubland. *Sci. Total Environ*. 709, 136210. [doi:10.1016/j.scitotenv.2019.136210](https://doi.org/10.1016/j.scitotenv.2019.136210).

Jia, X., Zha, T., Gong, J., Wang, B., Zhang, Y., Wu, B., et al. (2016). Carbon and water exchange over a temperate semi-arid shrubland during three years of contrasting precipitation and soil moisture patterns. *Agr. Forest Meteorol*. 228, 120-129. [doi:10.1016/j.agrformet.2016.07.007](https://doi.org/10.1016/j.agrformet.2016.07.007).

Kang, M., Zhang, Z., Noormets, A., Fang, X., Zha, T., Zhou, J., et al. (2015). Energy partitioning and surface resistance of a poplar plantation in northern China. *Biogeosciences* 12(14), 4245-4259. doi:10.5194/bg-12-4245-2015.

Kato, T., Tang, Y., Gu, S., Hirota, M., Du, M., Li, Y., et al. (2006). Temperature and biomass influences on interannual changes in CO_2_ exchange in an alpine meadow on the Qinghai-Tibetan Plateau. *Glob. Change Biol*. 12(7), 1285-1298. [doi:10.1111/j.1365-2486.2006.01153.x](https://doi.org/10.1111/j.1365-2486.2006.01153.x).

Li, C. (2012). *The research of correlativity between North subtropical secondary oak forest ecosystem carbon budget and asymmetrical environmental factors* (Nanjing, Jiangsu, China: MS Thesis of Nanjing Forestry University).

Li, H., Wang, A., Yuan, F., Guan, D., Jin, C., Wu, J., et al. (2016). Evapotranspiration dynamics over a temperate meadow ecosystem in eastern Inner Mongolia, China. *Environ. Earth Sci.* 75(11), 1-11. doi:10.1007/s12665-016-5786-z.

Li, H., Zhang, F., Li, Y., Wang, J., Zhang, L., Zhao, L., et al. (2016). Seasonal and inter-annual variations in CO_2_ fluxes over 10 years in an alpine shrubland on the Qinghai-Tibetan Plateau, China. *Agr. Forest Meteorol*. 228, 95-103. [doi: 10.1016/ j.agrformet.2016.06.020](https://doi.org/10.1016/j.agrformet.2016.06.020).

Li, H., Zhang, F., Wang, W., Li, Y., Lin, L., Wang, J., et al. (2018). The strongest EI Niño event stimulated ecosystem respiration, not evapotranspiration, over a humid alpine meadow on the Qinghai-Tibetan Plateau. *Ecol. Indic*. 91, 562-569. [doi:10.1016/j.ecolind.2018.04.039](https://doi.org/10.1016/j.ecolind.2018.04.039).

Li, R., Fan, Y., Feng, P., Song, Z., Li, X., Yan, H., et al. (2020). Net ecosystem carbon exchange and its affecting factors in a deciduous broad-leaved forest in Songshan, Beijing, China. *Chin. J. Appl. Eclo*. 31(11), 3621-3630. doi:[10.13287/j.1001-9332.202011.008](https://doi.org/10.13287/j.1001-9332.202011.008).

Liu, R., Cieraad, E., Li, Y., Ma, J. (2016). Precipitation pattern determines the inter-annual variation of herbaceous layer and carbon fluxes in a phreatophyte-dominated desert ecosystem. *Ecosystems* 19(4), 601-614. doi:10.1007/s10021-015-9954-x.

Liu, X., Li, Y., Chen, X., Zhou, G., Cheng, J., Zhang, D., et al. (2015). Partitioning evapotranspiration in an intact forested watershed in southern China. *Ecohydrology* 8(6), 1037-1047. [doi:10.1002/eco.1561](https://doi.org/10.1002/eco.1561).

Liu, Y., Zhou, G., Du, H., Berninger, F., Mao, F., Li, X., et al. (2018). Response of carbon uptake to abiotic and biotic drivers in an intensively managed Lei bamboo forest. *J. Environ. Manage*. 223, 713-722. doi:10.1016/ j. jenvman. 2018.06.046.

Liu, Y. (2018). *Analysis of the Dynamics and Driving Forces of Carbon Budget in Two Typical Bamboo (Phyllostachys edulis and Phyllostachys praecox) Forest Ecosystems* (Linan, Zhejiang, China: MS Thesis of Zhejiang A & F University).

Ma, J., Zha, T., Jia, X., Tian, Y., Bourque, C. P. A., Liu, P., et al. (2018). Energy and water vapor exchange over a young plantation in northern China. *Agr. Forest Meteorol*. 263, 334-345. doi:10.1016/j.agrformet.2018.09.004.

Ma, J., Jia, X., Zha, T., Bourque, C. P. A., Tian, Y., Bai, Y., et al. (2019). Ecosystem water use efficiency in a young plantation in Northern China and its relationship to drought. *Agr. Forest Meteorol*. 275, 1-10. [doi: 10.1016/j.agrformet.2019.05. 004](https://doi.org/10.1016/j.agrformet.2019.05.004)

Ma, N., Zhang, Y., Guo, Y., Gao, H., Zhang, H., and Wang, Y. (2015). Environmental and biophysical controls on the evapotranspiration over the highest alpine steppe. *J. Hydrol*. 529, 980-992. [doi:10.1016/j.jhydrol.2015.09.013](https://doi.org/10.1016/j.jhydrol.2015.09.013).

Niu, X., Jiang, H., Zhang, J., Fang, C., Chen, X., and Sun, H. (2016). Characteristics of CO₂ flux in an old growth mixed forest in Tianmu Mountain, Zhejiang, China.  *Chin. J. Appl. Ecol*. 27(1), 1-8, doi:10.13287/j.1001-9332.201601.010.

Niu, X., Liu, X., Liu, S., and Sun, P. (2018). Energy balance characteristics of a natural oak forest (Quercus aliena) at a transitional area from a subtropical to warm temperate climate, China. *Acta Ecol. Sin*. 38(18): 6701-6711. doi:10.5846 /stxb201803290650.

Ping, Y., Qiang, Z., Yang, Y., Zhang, L., Zhang, H., Hao, X., et al. (2018). Seasonal and inter-annual variability of the Bowen smith ratio over a semi-arid grassland in the Chinese Loess Plateau. *Agr. Forest Meteorol*. 252, 99-108. [doi:10.1016/j.agrformet.2018.01.006](https://doi.org/10.1016/j.agrformet.2018.01.006).

Qi, C., Liu, X., Yan, Y., and Yuan, G. (2019). Water Budget of Abandoned Steppe in Semiarid Area on the Loess Plateau in Different Rainfall Pattern Years. *Res. Soil. Water Conserv*. 26(1): 106-112. doi: 10.13869/j.cnki.rswc.2019.01.014.

Qiu, L., Zu, Y.G., Wang, W.J., Sun, W., Su, D.X., and Zheng, G.Y. (2011). CO_2_ flux characteristics and their influence on the carbon budget of a larch plantation in Maoershan region of Northeast China. *Chin. J. Appl. Eclo*.22,1-8. doi:10.13287/ j.1001-9332.2011.0031.

Qu, L., Chen, J., Dong, G., Jiang, S., Li, L., Guo, J., et al. (2016). Heat waves reduce ecosystem carbon sink stren gth in a Eurasian meadow steppe. *Environ. Res*. 144, 39-48. doi10.1016/j.envres.2015.09.004.

Shang, L., Zhang, Y., Lyu, S., and Wang, S. (2016). Seasonal and inter-annual variations in carbon dioxide exchange over an alpine grassland in the eastern Qinghai-Tibetan Plateau. *PloS One* 11(11), e0166837. [doi:10](https://doi.org/10).1371/journal.pone.0166837.

Shao, C., Chen, J., Li, L., Dong, G., Han, J., Abraha, M., et al. (2017). Grazing effects on surface energy fluxes in a desert steppe on the Mongolian Plateau. *Ecol. Appl*. 27(2), 485-502. [doi:10.1002/eap.1459](https://doi.org/10.1002/eap.1459).

Song, Q., Braeckevelt, E., Zhang, Y., Sha, L., Zhou, W., Liu, Y., et al. (2017). Evapotranspiration from a primary subtropical evergreen forest in Southwest China. *Ecohydrology* 10(4), e1826. [doi.org/10.1002/eco.1826](https://doi.org/10.1002/eco.1826).

Song, Q., Tan, Z., Zhang, Y., Sha, L., Deng, X., Deng, Y., et al. (2014). Do the rubber plantations in tropical China act as large carbon sinks? *Iforest*. 7(1), 42. doi:[10.3 832/ifor0891-007](https://doi.org/10.3832/ifor0891-007).

Song, X., Chen, X., Zhou, G., Jiang, H., and Peng, C. (2017). Observed high and persistent carbon uptake by Moso bamboo forests and its response to environmental drivers. *Agr. Forest Meteorol*. 247, 467-475. [doi:10.1016/ j.agrformet. 2017.09.001](https://doi.org/10.1016/j.agrformet.2017.09.001).

Sun, J., Sun, X., Hu, Z., and Wang, G. (2020). Exploring the influence of environmental factors in partitioning evapotranspiration along an elevation gradient on Mount Gongga, eastern edge of the Qinghai-Tibet Platea, China. *J. Mt. Sci.* 17(2), 384-396. doi:10.1007/s11629-019-5687-1.

Sun, S., Che, T., Li, H., Wang, T., Ma, C., Liu, B., et al. (2019). Water and carbon dioxide exchange of an alpine meadow ecosystem in the northeastern Tibetan Plateau is energy-limited. *Agr. Forest Meteorol*. 275, 283-295. [doi:10.1016/j.agrformet.2019.06.003](https://doi.org/10.1016/j.agrformet.2019.06.003).

Tan, Z., Zhang, Y., Schaefer, D., Yu, G., Liang, N., and Song, Q. (2011). An old-growth subtropical Asian evergreen forest as a large carbon sink. *Atmos. Environ*. 45(8), 1548-1554. doi:10.1016/j.atmosenv.2010.12.041.

Tian, X., Zhang, L., Zhang, X., Chen, Z., Zhao, L., Li, Q., et al. (2020). Evapotranspiration characteristics of degraded meadow and effects of freezethaw changes in the Three-River Source Region. *Acta Ecol. Sin*. 40(16): 5649-5662. doi: 10.5846/stxb201909151910.

Tie, Q., Hu, H., Tian, F., and Holbrook, N. (2018). Comparing different methods for determining forest evapotranspiration and its components at multiple temporal scales. *Sci. Total Environ*. 633, 12-29. doi:10.1016/ j.scitotenv. 2018.03.082.

Tong, X., Zhang, J., Meng, P., Li, J., and Zheng, N. (2017). Environmental controls of evapotranspiration in a mixed plantation in North China. *Int. J. Biometeorol* 61(2), 227-238. doi:10.1007/s00484-016-1205-0.

Wang, L. (2020). *Impacts of climate change and shrubification on carbon cycle in desert steppe* (Yinchuan, Ningxia, China: MS Thesis of Ningxia University). doi: 10.27257/d.cnki.gnxhc.2020.000383.

Wang, H., Li, X., Xiao, J., Ma, M., Tan, J., Wang, X., et al. (2019). Carbon fluxes across alpine, oasis, and desert ecosystems in northwestern China: The importance of water availability. *Sci. Total Environ*. 697, 133978. [doi:10.1016/j.scitotenv.2019. 133978](https://doi.org/10.1016/j.scitotenv.2019.133978).

Wang, L., Liu, H., Shao, Y., Liu, Y., and Sun, J. (2018). Water and CO_2_ fluxes over semiarid alpine steppe and humid alpine meadow ecosystems on the Tibetan Plateau. *Theor. Appl. Climatol*. 131(1): 547-556. doi:10.1007/s00704-016-1997-1.

Wang, L., Liu, H., Sun, J., and Shao, Y. (2017). Biophysical effects on the interannual variation in carbon dioxide exchange of an alpine meadow on the Tibetan Plateau. *Atmos. Chem. Phys*. 17(8): 5119-5129. doi:10.5194/acp-17-5119-2017.

Wang, H., Saigusa, N., Zu, Y., Wang, W., Yamamoto, S., and Kondo, H. (2008). Carbon fluxes and their response to environmental variables in a Dahurian larch forest ecosystem in northeast China. *J. Forestry Res*. 19(1), 1-10. doi:10.1007/s11676-008-0001-z.

Wu, J., Wu, H., Ding, Y., Qin, J., Li, H., Liu, S., et al. (2020). Interannual and seasonal variations in carbon exchanges over an alpine meadow in the northeastern edge of the Qinghai-Tibet Plateau, China. *PloS One* 15(2), e0228470. doi:10.1371/ journal. pone.0228470.

Xie, J., Zha, T., Zhou, C., Jia, X., Yu, H., Yang, B., et al. (2016). Seasonal variation in ecosystem water use efficiency in an urban-forest reserve affected by periodic drought. *Agr. Forest Meteorol*. 221, 142-151. [doi:10.1016/j.agrformet.2016.02. 013](https://doi.org/10.1016/j.agrformet.2016.02.013).

Xin, H., Wang, K., Yang, X., Zheng, X., and Chen, Q. (2014). The Variation Characteristics of Surface Energy Budget and Evapotranspiration over a Summer Grazed Semi-arid Grassland. *J. Chengdu Univ. Inf. Technol*. 29(05):539-546. doi: 10.16836/j.cnki.jcuit.2014.05.015.

Xu, M., Wang, H., Wen, X., Zhang, T., Di, Y., Wang, Y., et al. (2017). The full annual carbon balance of a subtropical coniferous plantation is highly sensitive to autumn precipitation. *Sci. Rep*. 7(1), 1-12. doi:10.1038/s41598-017-10485-w.

Yan, C., Zhao, W., Wang, Y., Yang, Q., Zhang, Q., and Qiu, G. (2017). Effects of forest evapotranspiration on soil water budget and energy flux partitioning in a subalpine valley of China. *Agr. Forest Meteorol*. 246, 207-217. [doi:10.1016/j.agrformet.20 17. 07.002](https://doi.org/10.1016/j.agrformet.2017.07.002).

Yan, J., Liu, X., Tang, X., Yu, G., Zhang, L., Chen, Q., et al. (2013). Substantial amounts of carbon are sequestered during dry periods in an old-growth subtropical forest in South China. *J. Forest Res*. 18(1), 21-30. doi:10.1007/s10310-012-0363-0.

Yang, Z., Zhang, Q., and Hao, X. (2019). Environmental and biological controls on monthly and annual evapotranspiration in China’s Loess Plateau. Theor. Appl. Climatol. 137(3), 1675-1692. doi:10.1007/s00704-018-2701-4.

Zhang, H., and Dou, R. (2020). Interannual and seasonal variability in evapotranspiration of alpine meadow in the Qinghai-Tibetan Plateau. *Arab. J. Geosci*. 13(18), 1-8. doi:10.1007/s12517-020-06022-1.

Zhang, J., Wang, J., and Gao, T. (2016). Observational study on evapotranspiration vorticity correlation flux in Xilamuren Grassland. *Inner Mongolia Water Resour*. (08): 11-12.

Zhao, H., Jia, G., Wang, H., Zhang, A., and Xu, X. (2019). Seasonal and interannual variations in carbon fluxes in East Asia semi-arid grasslands. *Sci. Total Environ*. 668, 1128-1138. doi: 10.1016/j.scitotenv.2019.02.378.

Zhang, T., Zhang, Y., Xu, M., Zhu, J., Chen, N., Jiang, Y., et al. (2018). Water availability is more important than temperature in driving the carbon fluxes of an alpine meadow on the Tibetan Plateau. *Agr. Forest Meteorol*. 256: 22-31. [doi:10.1016/j.agrformet.2018.02.027](https://doi.org/10.1016/j.agrformet.2018.02.027).

Zhang, T., Zhang, Y., Xu, M., Zhu, J., Wimberly, M., Yu, G., et al. (2015). Light-intensity grazing improves alpine meadow productivity and adaption to climate change on the Tibetan Plateau. *Sci. Rep*. 5(1), 1-12. doi:10.1038/srep15949.

Zhao, W., Liu, B., Chang, X., Yang, Q., Yang, Y., Liu, Z., et al. (2016). Evapotranspiration partitioning, stomatal conductance, and components of the water balance: A special case of a desert ecosystem in China. *J. Hydrol.* 538, 374-386. doi: 10.1016/j.jhydrol.2016.04.042.

Zhang, X. (2010). *Research on evapotranspiration and water use efficiency in secondary oak forest* (Nanjing, Jiangsu, China: MS Thesis of Nanjing Forestry University).

Zhang, X., Jin, C., Guan, D., Wang, A., Wu, J., and Yuan, F. (2012). Long-term eddy covariance monitoring of evapotranspiration and Its environmental factors in a temperate mixed forest in northeast China. *J. Hydrol. Eng*. 17(9), 965-974. [doi: 10.1061/(ASCE)HE.1943-5584.0000549](https://doi.org/10.1061/(ASCE)HE.1943-5584.0000549).

Zhang, Y., Tan, Z., Song, Q., Yu, G., and Sun, X. (2010). Respiration controls the unexpected seasonal pattern of carbon flux in an Asian tropical rain forest. *Atmos. Environ*. 44(32), 3886-3893. doi:10.1016/ j.atmosenv. 2010.07.027.

Zhang, Y., Zhu, W., Sun, X., and Hu, Z. (2018). Carbon dioxide flux characteristics in an Abies fabri mature forest on Gongga Mountain, Sichuan, China. *Acta Ecol. Sin*. 38(17): 6125-6135. doi:10.5846 / stxb201709051599.

Zhao Z., Zhang L., Kang W., Tian D., Xiang W., Yan W., et al. (2011). Characteristics of CO_2_ flux in a Chinese Fir plantation ecosystem in Huitong County, Hunan Province. *Sci. Silvae Sin*. 47(11): 6-12.

Zhu, G., Lu, L., Su, Y., Wang, X., Cui, X., Ma, J., et al. (2014). Energy flux partitioning and evapotranspiration in a sub‐alpine spruce forest ecosystem. *Hydrol. Process* 28(19), 5093-5104. [doi:10.1002/hyp.9995](https://doi.org/10.1002/hyp.9995).
